# Automatic ECG Quality Assessment Techniques: A Systematic Review

**DOI:** 10.3390/diagnostics12112578

**Published:** 2022-10-24

**Authors:** Kirina van der Bijl, Mohamed Elgendi, Carlo Menon

**Affiliations:** Biomedical and Mobile Health Technology Lab, ETH Zurich, 8008 Zurich, Switzerland

**Keywords:** electrocardiogram (ECG), signal quality indexes (SQIs), quality assessment, artefact detection, ambulatory monitoring, physiological signals

## Abstract

Cardiovascular diseases are the leading cause of death, globally. Stroke and heart attacks account for more than 80% of cardiovascular disease-related deaths. To prevent patient mismanagement and potentially save lives, effective screening at an early stage is needed. Diagnosis is typically made using an electrocardiogram (ECG) analysis. However, ECG recordings are often corrupted by different types of noise, degrading the quality of the recording and making diagnosis more difficult. This paper reviews research on automatic ECG quality assessment techniques used in studies published from 2012–2022. The CinC11 Dataset is most often used for training and testing algorithms. Only one study tested its algorithm on people in real-time, but it did not specify the demographic data of the subjects. Most of the reviewed papers evaluated the quality of the ECG recordings per single lead. The accuracy of the algorithms reviewed in this paper range from 85.75% to 97.15%. More clarity on the research methods used is needed to improve the quality of automatic ECG quality assessment techniques and implement them in a clinical setting. This paper discusses the possible shortcomings in current research and provides recommendations on how to advance the field of automatic ECG quality assessment.

## 1. Introduction

In 2019, cardiovascular diseases resulted in the death of 17.9 million people, worldwide, accounting for 32% of all deaths that year [1]. The number of deaths from cardiovascular diseases is expected to further increase to 23.6 million per year in 2030, due to the aging of the global population [1]. An electrocardiogram (ECG) is a simple non-invasive method for evaluating heart health. Thus, ECG signal analysis is a popular tool for diagnosing and preventing cardiovascular disease. ECG recordings are often corrupted by noise that resembles ECG waveforms. To prevent this, experts need to record the ECGs. Then, they need to manually check the ECG recordings to find high-quality subsections suitable for analysis. This time-consuming task requires the training and deployment of medical staff. This is especially problematic in developing countries where the number of trained medical staff is scarce in rural areas. In the last 10 years, the popularity of mHealth technologies has grown; this has given non-experts access to medical recording technologies that were previously only available to experts. Furthermore, developments in ECG device hardware have increased the ease and popularity of long ECG recording sessions conducted outside of hospital settings. Current ambulatory ECG tests often take 24, 48, or 72 h. This is done using a Holter monitor that records 12-lead ECGs [2]. Recordings up to 7–14 days or longer can be done using Personal ECG Monitors (PEMs) [3]. These devices produce one or three lead recordings. The longer recording time of PEMs in comparison to Holter devices increases the potential of detecting an arrhythmic event and providing a proper diagnosis [3]. Both the Holter monitor and PEM produce large quantities of ECG data that need to be quality checked before being used for diagnostic analysis. This requirement threatens to flood the medical system with low-quality data. To solve this problem, the quality of the collected data needs to be checked to ensure that low-quality data are removed before being sent for analysis.

Automatic ECG quality assessment techniques have been developed to ensure the quality of ECG recordings [4]. Most of this research was conducted after the PhysioNet/Computing in Cardiology Challenge 2011 [4]. This challenge aimed to make ECG assessments more accessible to patients that do not have access to an expert trained in ECG evaluating recordings. This was done by creating a smartphone algorithm that could assess the quality of an ECG recording while the patient is still present [4]. The algorithm should be able to perform the assessment in near-real time, which enables laypeople to perform high-quality ECG screenings and obtain ECG recordings without needing experts to assess the quality of the recording [4]. It also enables possible re-recordings on the same day when the patient is still present. This is especially useful for patients who live far from the medical clinic and might not be able return another day for a follow-up.

This paper reviews the relevant literature published between January 2012 and January 2022 that focuses on automatic ECG quality assessment techniques using machine learning and deep learning methods. It discusses the datasets that are commonly used, the types of algorithms, and the performance of the algorithms. It also evaluates the limitations and strengths of numerous papers and offers recommendations for future research.

## 2. Methods

### 2.1. Literature Search

**Figure 1:**
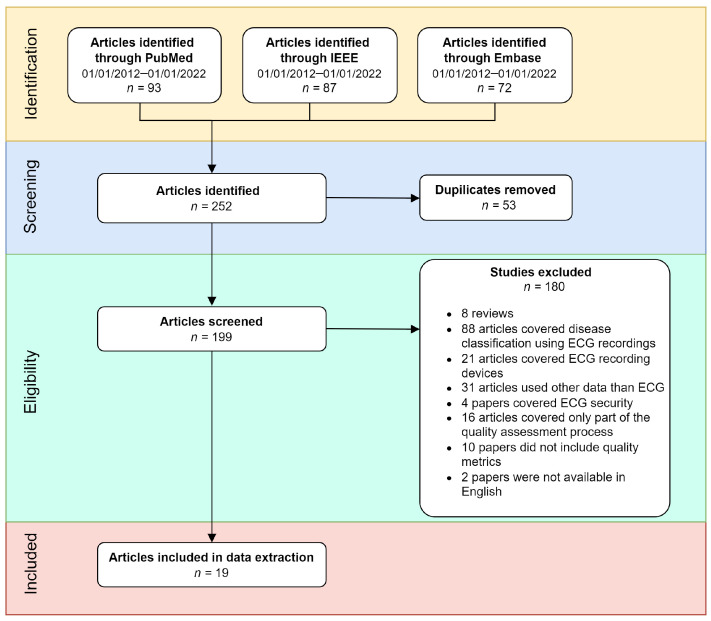
Flow diagram of the review methodology. From the initial search, a total of 252 studies were identified; of these, 53 studies were duplicates, 180 studies were excluded, and 19 studies were included in the analysis.

A literature search was performed using the PubMed, IEEE, and Embase databases. The results were filtered for the period of January 2012 to January 2022. After analysis, the search resulted in 19 publications that addressed automatic ECG quality assessment. The exclusion criteria are shown in Figure 1. The following search terms were used: (ECG OR electrocardiogram) AND (signal quality) AND (index OR method OR algorithm) AND (machine learning OR deep learning).

### 2.2. Inclusion and Exclusion Criteria

The inclusion and exclusion criteria were used to ensure that only papers on automatic ECG quality assessment techniques were included in the analysis. As shown in Figure 1, 252 papers were initially identified using the previously mentioned search terms. Studies were excluded if they were duplicates (n = 53), if they were reviews (n = 8), if they covered disease classification (n = 88), if they covered ECG recording devices (n = 21), if they used other data than ECG (n = 31), if they covered ECG security (n = 4), if they only covered a part of the quality assessment process (n = 16), if they did not include a quality metric (n = 10), or if they were not available in English (n = 2). Some classification papers covered noise classification methods from ECG recordings. However, they did not include metrics to assess the quality of the noise removal, so they were omitted from the analysis.

## 3. Results

The sensitivity, specificity, and accuracy were calculated with Equations (1)–(3), respectively. Here, TP is the number of true positives (acceptable quality ECG recordings classified as acceptable), FP is the number of false positives (acceptable quality ECG recordings classified as unacceptable), TN is the number of true negatives (unacceptable quality ECG recordings classified as unacceptable), and FN is the number of false negatives (acceptable quality ECG recordings classified as unacceptable). Sensitivity refers to the TP. It calculates the percentage of positive results that were supposed to be positive. Specificity refers to the TN rate. It calculates the percentage of negative results that were supposed to be negative.

(1) $$\mathrm{Sensitivity}=\frac{\mathrm{TP}}{\mathrm{TP}\;+\;\mathrm{FN}}$$

(2) $$\mathrm{Specificity}=\frac{\mathrm{TN}}{\mathrm{TN}\;+\;\mathrm{FP}}$$

(3) $$\mathrm{Accuracy}=\frac{\mathrm{TP}\;+\;\mathrm{TN}}{\mathrm{TP}\;+\;\mathrm{FP}\;+\;\mathrm{TN}\;+\mathrm{FN}}$$

The area under the curve (AUC) refers to the area under the receiver operator characteristic (ROC). The ROC is a plot showing the performance of the model at different classification thresholds. It plots sensitivity on the *x-axis* and 1-specificity on the *y-axis*, as shown in Equation (4). The AUC measures the area under this curve to create a value ranging between 0 and 1. The AUC is 0 if all the predictions of a model are incorrect, no matter the threshold. The AUC is 1 if all the predictions of a model are correct, no matter the threshold.

(4) AUC=<Sensitivity,1−Specificity>

Table 1 shows a summary of the data gathered on the different papers. All the papers included in the analysis used a binary classification system. The ’Leads’ column refers to the number of leads per label used in the analysis. Thus, for 12 leads, the data has one label per 12 leads.

**Figure 2:**
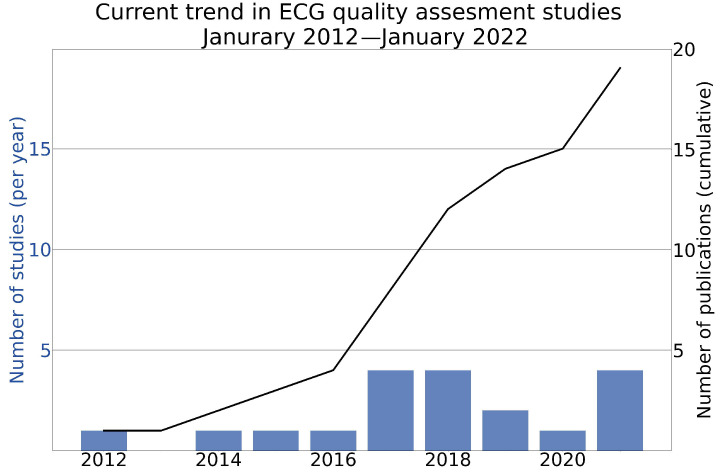
Cumulative sum of the publications and the total number of studies that addressed automatic ECG quality assessment techniques from January 2012 to January 2022.

Figure 2 shows the cumulative sum of the publications and the total number of studies published per year that addressed automatic ECG quality assessment. The findings show that the number of publications was low in 2012–2016. There was an exponential increase in the number of publications as of 2017.

**Figure 3:**
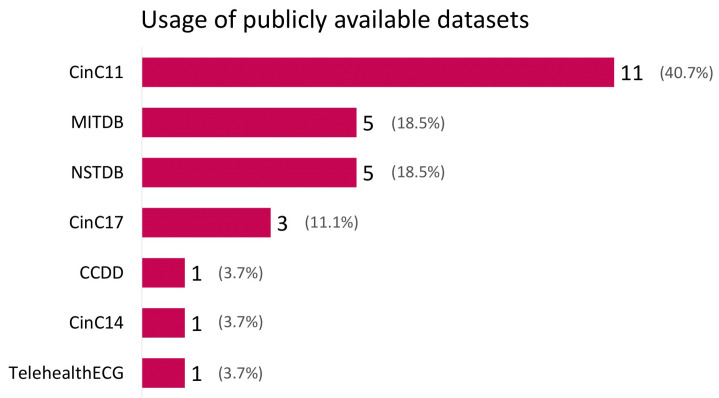
Bar plot of the publicly available datasets used by studies published between January 2012 and January 2022. The dataset most used was CinC11 [4]. The percentages represent the percentage of the dataset out of the total number of datasets used in the studies.

Figure 3 shows the number of times different publicly available datasets were used in the reviewed studies. Private datasets are not shown. The most used dataset was CinC11 [4], it was used in 11 studies. MITDB [15] is also known as the MIT-BIH Arrhythmia Database [15]. It was used in five studies; it is the ECG dataset that is most often used after CinC11 [4]. NSTDB [13] is the only dataset without ECG recordings; it only includes noise commonly present in ECG recordings. It was used in five studies. Cinc17 [7] was used in three studies. The CCDD [21], CinC14 [17], and TELE ECG [18] datasets were all used in one study. Seven publicly available datasets were used 27 times. Thus, some of the 19 studies discussed in this review used more than one publicly available dataset in their analysis.

**Figure 4:**
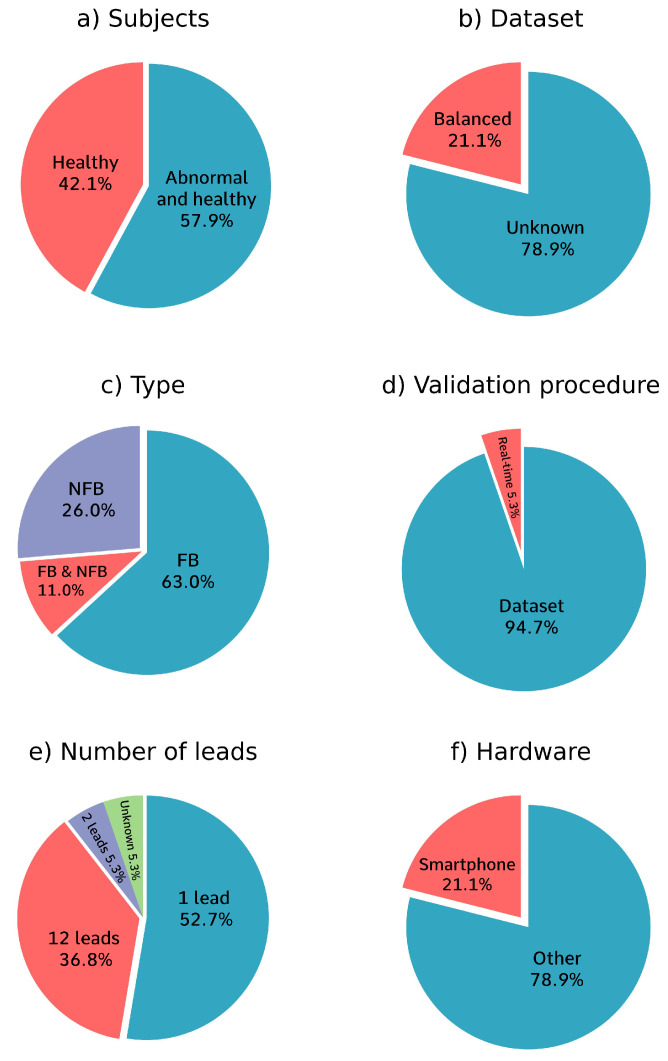
Pie charts of the analysis methods used. (**a**) 57.9% of the papers used data from both healthy and diseased subjects for training and 42.1% only used data from healthy subjects without heart conditions for training. (**b**) 21.1% of the papers balanced their data; 78.9% of the papers did not mention whether they used data balancing techniques. (**c**) 63.0% of the papers used an FB (feature-based, machine learning) algorithm, 26.0% of papers used an NFB (non-feature-based, deep learning) algorithm, and 11.0% of the papers used a combination of both FB and NFB algorithms. (**d**) Only one paper validated the algorithm that was created in real-time with test subjects. (**e**) 47.4% of the papers used 1-lead data, 36.8% of the papers used 12-lead data, one paper used 2-lead data, one paper used both 1-lead and 5-lead data, and one paper did not mention the number of leads used. (**f**) 21.1% of the papers mentioned that their algorithm is meant to run on a smartphone.

Figure 4 shows four pie charts with information on the different analysis methods used. Figure 4a refers to the training data. It shows that 42.1% of the papers only used ECG recordings from healthy subjects to train their models and 57.9% of the papers also included ECG recordings from people with abnormal heart rhythms for training. Figure 4b shows that 21.1% of the papers overcame class imbalances in the data by re-balancing the dataset. However, 78.9% of the papers did not mention whether they used balancing techniques.

Figure 4c shows that 63.0% of the papers used a purely feature-based (FB) approach, that 26.0% of the papers used a purely non-feature-based (NFB) approach, and 11.0% of the papers used both FB and NFB methods in their algorithm. Figure 4d shows that only one paper tested their algorithm in real-time using 20 test subjects [23]. That study did not include the participants’ health status and demographic data. The rest of the papers only tested their algorithm on one dataset. The eventual goal is to create an algorithm that works in real-time, so research papers should also strive to do that.

Figure 4e shows that most of the papers evaluated the quality of the ECG recordings per a single lead, while 37% of the papers evaluated the quality of the ECG recordings per 12 leads, which is the number of leads collected in most standard ECG tests, and one paper used a different approach and evaluated the quality of 2-lead ECG recordings, which is used for ambulatory ECG recordings [14], one paper did not mention the number of leads that were used [23].

Figure 4f shows that 21% of the papers mentioned that their algorithm was meant to run on a smartphone. The rest of the papers used a device other than a smartphone to run their algorithm.

## 4. Discussion

### 4.1. Common Algorithms and Methods Used in ECG Quality Assessment

ECG quality assessment technologies can be divided into two broad categories: FB and NFB.

**Figure 5:**
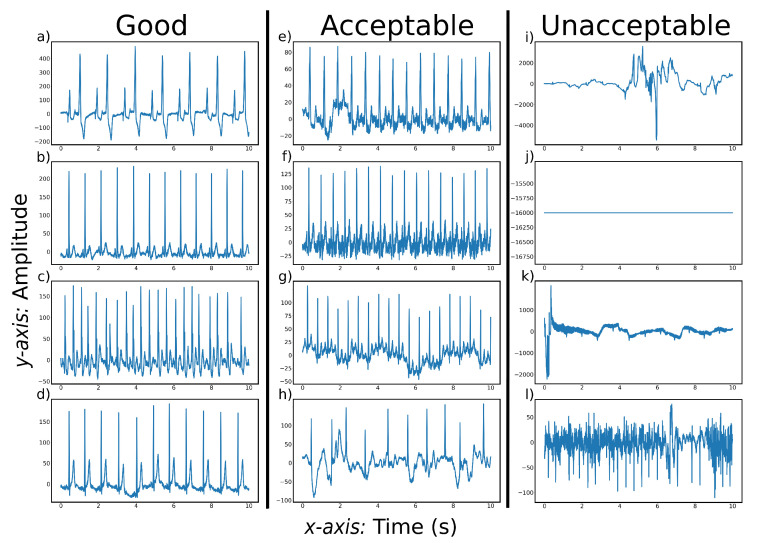
Typical examples of CinC11 [4] signals at three quality levels. (**a**–**d**) show ECG recordings with regular beats of good quality. (**e**–**h**) show ECG recordings of acceptable quality that can become good quality after preprocessing. (**i**–**l**) show ECG recordings of unacceptable quality.

As shown in Figure 4c, 63% of the papers used a purely FB approach and 11% of the papers used both FB and NFB methods in their algorithm. FB ECG quality assessment algorithms often use a stepwise approach to incrementally check the quality of a lead. At every step, different aspects of the lead are quality checked. The lead is classified as ‘unacceptable’ if the lead is deemed bad quality at one of the quality assessment steps. The lead is classified as ’acceptable’ if the lead passes all the quality checks. A commonly used first step is to reject recordings that have a missing lead [5,16,23]. Figure 5j shows an example of a recording where the lead is missing. This is an easily identifiable characteristic of the ECG data and is often chosen as the first assessment step. This works well for 1-lead data. However, in multi-lead data it is questionable if one flat lead is a good reason to reject all the leads. Not all leads are needed to diagnose heart disease. If the quality of the rest of the leads is high, it could be advantageous to retain the recording even though a lead is missing, especially in situations where it is difficult to collect high-quality data, such as when the patient is moving. After checking for missing leads, the common quality checking methods and their order diverge.

ECG quality assessment technologies can be divided into two broad categories: FB (machine learning) methods and NFB (deep learning) methods. As shown in Figure 4c, 63% of the papers used a purely FB approach and 11% of the papers used both FB and NFB methods in their algorithm. FB ECG quality assessment algorithms often use a stepwise approach to incrementally check the quality of a lead. Often, the included methods check for background noise, beat consistency, amplitude range, and QRS detection. Whether an ECG lead needs to be removed due to background noise depends on the type and source of the background noise. Figure 5l shows an example of a recording with an unacceptable level of background noise.

EMG interference is a type of noise that can decrease the quality of an ECG recording. Figure 5f shows a recording with EMG interference that stems from power line interference. This is relatively easy to remove from the recording. Less predictable sources of EMG interference are more difficult to remove and can be the reason the ECG recording is rejected. For example, this can be caused by muscle contractions that produce EMG signals, or vibrations caused by speaking [9,12,23].

The beat consistency assessment checks whether there are any unexpected events in the recording. On average, the next heartbeat will look like the previous one. A big shift in the shape of the ECG recording likely means that there is an artifact in the data [12]. A low signal-to-noise ratio is a reason for rejecting the ECG recording, especially if the noise is greater than the amplitude range that a heart can produce [23]. This can potentially be problematic because not all sudden changes are due to noise. Diagnostically relevant information can be misclassified as noise and make the diagnosis more difficult.

QRS detection is often the final step of ECG quality assessment algorithms. A QRS complex is the electrical current that is caused by the depolarization of the right and left ventricles of the heart. The QRS complex is the largest characteristic of the heart in an ECG recording and the easiest to identify. If the QRS complex cannot accurately be found, the ECG recording will be rejected [28]. As is the case with artifact identification, diagnostically relevant information may be misclassified as noise. The QRS complex may be absent in diagnostically relevant situations like ventricular fibrillation.

As shown in Figure 4c, 26% of the papers used a purely NFB approach and 11% of the papers included both FB and NFB methods in their algorithm. NFB ECG quality assessment technologies use both custom and pre-trained models. They feed labelled data to the model, which then learns to recognize features of the data. An advantage of this approach is that the NFB model could learn to classify the ECG recordings based on characteristics that would not have been included when using an FB model. This can lead to a better algorithm performance. This is also a disadvantage of NFB models in ECG quality assessment. It is difficult to diagnose why an ECG recording is rejected due to the ‘black box’ nature of NFB models. This makes it difficult for the person conducting the ECG recording to receive feedback on what is causing the ECG recording to be rejected.

### 4.2. Risk Bias

As shown in Figure 3, the CinC11 [4] dataset was most frequently used in the studies; it originated from the PhysioNet/Computing in Cardiology Challenge 2011. Eleven studies only cross-validated their results on the same dataset that they used to train their model [6,8,10,11,12,14,19,22,24,25,29]. This likely results in a higher accuracy than cross-validating on data gathered separately from the training set. This can result in high accuracy scores because the model does not generalize to unseen data.

Figure 3 shows that five papers used the NSTDB database to create noisy data. The NSTDB database is 30 min of noise commonly present in ECG recordings. It only contains three types of noise: baseline wander, muscle artifacts, and electrode motion artifacts. This database is limited in diversity and size. This results in noise segments having to be reused in long recordings and relatively one-sided training data. This can result in models whose predictions do not transfer well to real-life situations, where more types of noise are present.

All the reviewed papers assessed their results using either sensitivity, specificity, and accuracy metrics or the AUC. These metrics work well for balanced datasets, but they can be unreliable when the dataset is unbalanced [30]. A ‘balanced’ dataset is one with equally represented training and testing. In the case of ECG quality assessment, this refers to the amount of data in the acceptable and unacceptable quality classes of the ECG data. When these quality classes are unbalanced, the results of an algorithm can seem better than they actually are when using the sensitivity, specificity, accuracy, or AUC metrics [30].

When the dataset is unbalanced, the Matthews correlation coefficient (MCC) can evaluate the performance more reliably than sensitivity, specificity, accuracy, and AUC [30]. None of the papers used the MCC to evaluate the performance of their algorithm. As shown in Figure 4b, 21% of the papers mention that they balanced the dataset [6,12,19,29]. The rest of the papers did not mention whether they balanced the dataset or whether they used other methods to overcome class imbalances.

Gender bias could be a problem for ECG quality assessment as the PR interval, heart rate, QRS duration and lead voltages show gender-related differences [31]. The difference in ECGs between genders is most significant in anterior leads for people under the age of 40 [32]. An algorithm without gender bias would not rely on gender-specific metrics, or it would customize the thresholds of the metrics based on the gender of the patient whose ECG is being recorded [32]. ECG features that are not gender-specific include ST elevation and reciprocal ST depression, and/or T wave inversion [32].

Careless dataset balancing to remove class imbalances can lead to other imbalances in the dataset. For example, the CinC11 dataset has an even male/female ratio in its data. This even ratio can be removed by re-balancing the dataset to have an even acceptable/unacceptable ratio. This can inadvertently lead to a gender bias in the algorithm when the number of male and female recordings in the re-balanced dataset is not checked. This type of unbalanced data is more difficult to detect because it does not show up in standard performance evaluations of the algorithm and can provide misleading results [33].

The goal is to use ECG quality assessment algorithms in real-life situations, which is more unpredictable than when using the algorithms with a dataset. As shown in Figure 4d, only one of the studies analyzed in this review validated the performance of its algorithm on human subjects [23]. The different datasets used and the difference between the datasets and real-life testing increases the difficulty of comparing the results of different studies. This creates a distorted view of the performance of the different algorithms and leads to an under-appreciation of algorithms tested on more difficult data. More difficult data could be data with multiple noise sources. With more different types of noise, the model is less likely to overfit on one specific type of noise and is more likely to learn the characteristics of acceptable ECG recordings and how they differ from unacceptable ECG recordings. For example, it is easy to identify electrode loss, which results in a flat horizontal line on the ECG, as noise. Electrode loss as noise would be effective to use as training data because the model can easily learn that a flat line equals noise without learning anything about the characteristics of acceptable ECG data.

### 4.3. Code Availability

None of the papers in this review published the code they used for their algorithms. It is impossible to validate the results of the studies without having access to the code used to produce the results. Open-source code is important for advancing the field of ECG quality assessment and facilitating collaboration between research groups. Not publishing code hampers innovation and delays the deployment of ECG quality assessment techniques.

It is recommended that studies make their code open source so that other scientists can validate and improve on the research findings. If it is not possible to make the code open source, studies should at least include information on how the algorithm was structured. This includes what programming language was used, which formulas the algorithm used, and how the results were validated.

### 4.4. User-Friendliness

The goal of the ECG quality assessment algorithms is to make ECG quality assessment easier to conduct and available to laypeople. To accomplish this, the algorithms need to be easy to use and understand to ensure that inexperienced people can use them. None of the papers mention the user-friendliness of the algorithms they created. Furthermore, the algorithms need to have low computational demands to increase the accessibility of the automatic ECG assessment techniques. Preferably, it should also be possible to run the algorithms on smartphones, as this removes the need to use specialized hardware to run the algorithm. This makes it easier to record high-quality ECGs in places where specialized hardware or fast internet speed may not be available, but where people have access to smartphones. Alternatively, it could be possible to use a phone app that uses cloud computing to assess ECG quality. However, this requires access to the internet and cloud computing resources. This reduces the accessibility of the algorithm. The algorithms should also be fast enough to produce results while the patient is still present. This enables a re-recording if the quality of the previously recorded ECG is insufficient. This is mainly beneficial when patients live far from the clinic and cannot easily visit again for a re-recording. Of the papers included in this review, 21% mention that their algorithm is meant to run on a smartphone, but the technical specifications of the app are not mentioned [5,16,23,29]. Moreover, no fully functional apps have been developed.

### 4.5. Study Limitations

This study has some limitations. It only focused on publications on ECG quality assessment techniques in the scientific literature. It does not include private and open-source publications of ECG quality assessment techniques that were not in the scientific databases used for this research. Only research published between 2012 and 2022 was included to focus on recent publications. This means that possibly interesting publications from before 2012 were excluded. The studies analyzed in this review were limited by the availability of labelled datasets and the quality of the labels provided. Furthermore, most of the datasets had two or three labels and did not include information on the source of noise present in the ECG recordings. Information on the gender and medical history of the subject in the dataset was often not provided.

### 4.6. Recommendations for Future Advancements

The current studies on ECG quality assessment have made significant advances. However, it is recommended that future research should:

- Validate the algorithms in real-life conditions and, if test subjects are used, researchers should include demographic data, such as gender, age, and health status;
- Use class balancing techniques to generate unbiased sensitivity, specificity, and accuracy metrics, and check to determine if the demographic data, such as age and gender, are still balanced after class balancing is performed.
- Make the code for the algorithm open source so the results can be validated and the algorithm can be used for future research;
- Focus on developing algorithms that can be run on a smartphone; this requires including the computational demands of the algorithm or the kind of hardware it is meant to run on.

## 5. Conclusions

Cardiovascular diseases are the leading cause of death, worldwide. The number of people suffering from cardiovascular diseases is expected to increase due to the aging of the global population. ECG recordings are a common method used to identify cardiovascular disease. The quality of ECG recordings needs to be checked to facilitate the diagnosis of cardiovascular disease. Currently, this is done manually, which is time-consuming. Automatic ECG quality assessment can speed up the data collection and the diagnostic process of cardiovascular diseases. For this, automatic ECG quality assessment tools need to be easy for clinicians to use so the results can be more quickly analyzed. Currently, few studies have focused on conducting an automatic ECG quality assessment in real-world settings. Thus, more research is needed on how ECG quality assessment technologies can be deployed. Furthermore, studies need to incorporate more details on what methods they used to produce their results. This can help other researchers improve the research done on the quality of automatic ECG assessment techniques. Implementation of automatic ECG quality assessment techniques has the potential to transform cardiovascular disease diagnosis by making ECG recordings easier to perform and faster to analyze.

## Figures and Tables

**Table 1:** Summary of all the identified publications on ECG quality assessment techniques. Note that FB refers to feature-based and NFB refers to non-feature-based. N/R refers to not received, meaning that the value was not mentioned in the article. The values have been averaged for studies that reported multiple results. * Averaged value.

Name First Author and Year	Type	Data	Leads	Cross Validation	Sensitivity (%)	Specificity (%)	AUC (%)	Accuracy (%)
Fu et al. (2021) [5]	FB	Private dataset	1	N/R	97.41	86.50	N/R	95.63
Huerta et al. (2021) [6]	NFB	CinC17 [7]	1	N/R	90.00	91.00	N/R	90.00
Liu et al. (2021) [8]	NFB	CinC11 [4]	12	N/R	97.67	84.73	N/R	93.09
Xu et al. (2021) [9]	FB	Private dataset	1	N/R	N/R	N/R	N/R	95.23 *
Huerta et al. (2020) [10]	NFB	CinC17 [7]	1	N/R	90.37 *	93.00 *	N/R	87.77 *
Hermawan et al. (2019) [11]	FB	CinC11 [4]	1 + 5	N/R	92.00 *	67.00 *	N/R	85.75
Moeyersons et al. (2019) [12]	FB	CinC17 [7], NSTDB [13]	1	3-fold	98.37	96.43	99.67 *	N/R
Ansari et al. (2018) [14]	NFB	MITDB [15], NSTDB [13]	2	N/R	N/R	N/R	96.41	N/R
Liu et al. (2018) [16]	FB	CinC11 [4], CinC14 [17], TELE ECG [18], Private dataset	12	10-fold	89.08 *	88.15 *	N/R	97.15
Yaghmaie et al. (2018) [19]	FB	CinC17 [7], NSTDB [13], MITDB [15]	1	5-fold	96.20	97.70	93.18	96.90
Zhang et al. (2018) [20]	NFB	CinC11 [4], CCDD [21]	12	N/R	N/R	N/R	N/R	96.45
Athif et al. (2017) [22]	FB	CinC11 [4]	12	5-fold	91.20	91.60	N/R	91.10
Satija et al. (2017) [23]	FB	CinC11 [4], MITDB [15]	N/R	N/R	N/R	96.71	N/R	N/R
Taji et al. (2017) [24]	NFB + FB	MITDB [15], NSTDB [13]	1	4-fold	98.20	98.30	N/R	97.20
Xia et al. (2017) [25]	FB	CinC11 [4]	12	N/R	N/R	N/R	N/R	91.60
Orphanidou et al. (2016) [26]	FB	Private dataset	1	N/R	92.50 *	98.00 *	N/R	95.00 *
Morgado et al. (2015) [27]	FB	CinC11 [4]	12	10-fold	86.08 *	89.05 *	91.25 *	92.25
Li et al. (2014) [28]	FB	CinC11 [4], NSTDB [13], MITDB [15]	12	N/R	N/R	N/R	N/R	96.51 *
Clifford et al. (2012) [29]	NFB + FB	CinC11 [4], NSTDB [13]	1 + 12	N/R	89.00	99.00	N/R	97.00

## Data Availability

Not applicable.
